# Sustainable Desalination by 3:1 Reduced Graphene Oxide/Titanium Dioxide Nanotubes (rGO/TiONTs) Composite via Capacitive Deionization at Different Sodium Chloride Concentrations

**DOI:** 10.3390/nano9091319

**Published:** 2019-09-15

**Authors:** John Paolo L. Lazarte, Liza Bautista-Patacsil, Ramon Christian P. Eusebio, Aileen H. Orbecido, Ruey-an Doong

**Affiliations:** 1 Chemical Engineering Department, Malayan Colleges Laguna, Pulo-Diezmo Rd. Cabuyao City, Laguna 4025, Philippines; 2 Chemical Engineering Department, De La Salle University, 2401 Taft Avenue, Manila 1004, Philippines; aileen.orbecido@dlsu.edu.ph; 3 Chemical Engineering Department, University of the Philippines Los Baños, College, Laguna 4031, Philippines; rpeusebio@up.edu.ph; 4 Institute of Environmental Engineering, National Chiao Tung University, Hsinchu 30010, Taiwan; radoong@mx.nthu.edu.tw; 5 Department of Biomedical Engineering and Environmental Sciences, National Tsing Hua University, Sec. 2 Kuang-Fu Road, Hsinchu 30013, Taiwan

**Keywords:** reduced graphene oxide, titanium dioxide nanotubes, sodium chloride, sustainable desalination, capacitive deionization

## Abstract

The capability of novel 3:1 reduced graphene oxide/titanium dioxide nanotubes (rGO/TiONTs) composite to desalinate using capacitive deionization (CDI) employing highly concentrated NaCl solutions was tested in this study. Parameters such as material wettability, electrosorption capacity, charge efficiency, energy consumption, and charge-discharge retention were tested at different NaCl initial concentrations—100 ppm, 2000 ppm, 15,000 ppm, and 30,000 ppm. The rGO/TiONTs composite showed good material wettability before and after CDI runs with its contact angles equal to 52.11° and 56.07°, respectively. Its two-hour electrosorption capacity during CDI at 30,000 ppm NaCl influent increased 1.34-fold compared to 100 ppm initial NaCl influent with energy consumption constant at 1.11 kWh per kg with NaCl removed. However, the percentage discharge (concentration-independent) at zero-voltage ranged from 4.9–7.27% only after 30 min of desorption. Repeated charge/discharge at different amperes showed that the slowest charging rate of 0.1 A·g^−1^ had the highest charging time retention at 60% after 100 cycles. Increased concentration likewise increases charging time retention. With this consistent performance of a CDI system utilizing rGO/TiONTs composite, even at 30,000 ppm and 100 cycles, it can be a sustainable alternative desalination technology, especially if a low charging current with reverse voltage discharge is set for a longer operation.

## 1. Introduction

Water is a vital resource for domestic, commercial, and industrial activities. However, water resources that serve such purposes are depleting. Given the current population increase and human water consumption behavior, by 2030 there is an expected 40% water supply deficit [1]. This is projected to happen even though the Earth’s waters cover 71% of the planet. Aside from the exploitation of useable water sources, the problem is that 96.5% of the world’s available water comes from seas and oceans. Presently, this hindrance poses a challenge with respect to how these potential water sources can be utilized effectively in order to increase supply. For utilizing this huge reservoir, a promising solution is desalination. One of the most helpful technologies developed for this is membrane separation. Although they have made the separation of water from salts possible, most membrane processes are costly due to their high energy demand, making continuous production unsustainable. With this, there is a need to look for alternative processes available. This may be addressed by capacitive deionization, an emerging desalination technology. Capacitive deionization (CDI) is a technology that makes possible the removal of ions/salts from a solution through an electric field generated when the capacitor electrodes are charged. Its performance in salts removal, as reported in studies, is promising, and it can remove different kinds of ions such as fluorides, calcium, sodium, chloride, potassium, iron (III), magnesium, sulfate, bromide, and nitrate [2,3,4,5,6,7,8,9].

The capacity of CDI for desalination was first demonstrated during mid-1960s [9] and, since then, studies to improve its efficiency have been conducted. Among all parameters that can be examined for this technology to become sustainable, the most thoroughly studied are electrochemical properties, electrosorption removal efficiency, total dissolved salts concentration, and energy consumption [8,10]. Nowadays, capacitive deionization is known to have lower energy consumption than its membrane desalination counterparts—however, this is only true at low to brackish water salt concentration levels [9]. This gap that must be addressed in order to make capacitive deionization more efficient, which has led researchers to focus on testing different materials. Some of the common materials are based on hydrogels, activated carbon, and graphene. The electrosorption capacity of these materials ranges from 4.8–49.34 mg·g^−1^ [11,12,13,14,15,16,17,18,19,20,21,22]. The lowest electrosorption capacity recorded was obtained from CDI using plain mesoporous carbon of Gao et al. [12], while the highest electrosorption capacity came from CDI using graphene hydrogel of Ma, Wang, and Yu [21]. However, the electrosorption time varied from one study to another. For instance, the lowest electrosorption capacity of 4.8 mg·g^−1^ took three hours of electrosorption while the highest electrosorption capacity of 49.34 mg·g^−1^ took five hours. In this case, the performance would be hard to compare as it is logical that a higher electrosorption capacity will be obtained after a longer operation. The electrosorption capacities obtained cannot just be normalized with respect to the time elapsed as their behavior is not linear. Moreover, for studies involving saturation, such as CDI using carbon hydrogel of Ma et al. [21] and P-doped carbon nanofiber aerogels of Y. Li et al. [22], material performance could be comparable in terms of electrosorption capacity. However, in this case, the amount of time necessary to reach saturation becomes a relevant consideration. For the rest of studies that did not reach saturation, the only guarantee that one material is better than the other is a higher electrosorption capacity achieved within a shorter span of time. For instance, it is evident that the TiO_2_ nanorod-rGO used by El-Deen et al. [11] is better compared to the mesoporous carbon used by Gao et al. [12], highly porous activated carbon used by Feng et al. [15], and carbon black loaded N-doped carbon aerogel used by Rasines et al. [13]. However, with this electrosorption capacity, a huge amount of material would still be required to be able to complete seawater desalination. Furthermore, this kind of composite has only been proven stable at 1400 ppm [23]. However, due to its ability to attain an electrosorption capacity of 9.1 mg·g^−1^ in a short span of five minutes, an improved composite of rGO and TiO_2_ has become a promising consideration for this study.

Hence, there is a need to test an electrode material at seawater concentration using an rGO and TiO_2_ composite with a more improved anatase structure than the TiO_2_ nanorod-rGO, in order to make it highly capacitive and electrosorptive. This study used this novel material, a composite of reduced graphene oxide (rGO) and titanium dioxide nanotubes (TiONTs) with a mass ratio of 3:1, respectively, synthesized by Lazarte et al. [24]. It has already been found to be an effective material for capacitive deionization, Cu (II) and Pb (II) ions in particular [25]. With sodium chloride as influent, the potential for the sustainable desalination of 3:1 rGO/TiONTs composite was assessed based on its (1) surface properties—wettability in particular, (2) chronoamperometric response to constant voltage at different concentrations, (3) chronocoulometric response to constant current operation at different charge-discharge ratios and concentrations. These objectives were aimed at demonstrating that the 3:1 rGO/TiONTs composite can be a sustainable alternative to other desalination technologies.

## 2. Materials and Methods

### 2.1. Chemicals and Reagents

The following chemicals and reagents were used for synthesis of the composite: graphite (graphite powder, Aldrich, Milwaukee, WI, USA), sulfuric acid (95–98% H_2_SO_4_, Merck, Darmstadt, Germany), phosphoric acid (85% H_3_PO_4_, Sigma, St. Louis, MO, USA), potassium permanganate (KMnO_4_ powder, Merck, Darmstadt, Germany), hydrogen peroxide (30% H_2_O_2_, Riedel-de Haën, Sleeze, Germany), hydrochloric acid (36.5–38% HCl, J.T. Baker, Phillipsburg, NJ, USA), sodium borohydride (98% NaBH_4_, Aldrich, Milwaukee, WI, USA), titanium dioxide (TiO_2_ ST01 powder, Ishihara Sangyo, Tokyo, Japan), sodium hydroxide (99% NaOH pellets, J.T. Baker, Phillipsburg, NJ, USA), ethanol (99.5–98% C_2_H_6_O, Riedel-de Haën, Sleeze, Germany), deionized (DI) water, carbon black (carbon powder, Uni-onward Corp., New Taipei, Taiwan), N-methyl-2-pyrrolidone (>99% NMP, Sigma-Aldrich, Milwaukee, WI, USA), and polyvinylidenefluoride (PVDF, Aldrich, Milwaukee, WI, USA). For the solutions desalinated, sodium chloride (99.9% NaCl crystals, Ajax Finechem, Australia) was dissolved in deionized (DI) water. All reagents were used as received without further purification.

### 2.2. Synthesis of 3:1 Reduced Graphene Oxide/Titanium Dioxide Nanotubes (rGO/TiONTs)

The composite was synthesized by combining rGO and TiONTs in a microwave reactor for 3 h at 600 W initial power maintained at 150 °C. The composite consists of 3 parts rGO and 1 part TiONTs by mass. Pure reduced graphene oxide (rGO) was synthesized by adapting the procedure of Lazarte et al. [24].

The synthesis of rGO involves a two-step process conversion: from graphite to graphene oxide and conversion of graphene oxide to reduced graphene oxide. Conversion of graphite to graphene oxide lasted for 12 h at 45 °C. The reagents used for this reaction are graphite (2 g), H_2_SO_4_ (225 mL), H_3_PO_4_ (25 mL), and KMnO_4_ (6 g). The mixture was poured into 225 mL DI water and kept in an ice bath under constant stirring while slowly pouring 3 mL hydrogen peroxide. The solution was continuously stirred for 30 min. Afterwards, the product was washed until pH reached 5–7, and dried. The resulting film was then converted to reduced graphene oxide by another 12 h of reaction with sodium borohydride as reducing agent at 100 °C. After the reaction, the sample was then dried. On the other hand, TiONTs was synthesized by subjecting 300 mg TiO_2_ ST01 into 10 mL of 10 M NaOH. The reaction lasted 3 h at 150 °C under 600 W initial power. The sample was then dried. The synthesized pure rGO and TiONTs were stored separately until composite synthesis.

### 2.3. Reduced Graphene Oxide/Titanium Dioxide Nanotubes Surface Properties

Before the CDI process, the electrode coated on the titanium plate (current collector) was imaged using Dino-Lite Digital Microscope Premier and was subjected to a small drop of water to determine contact angle. This procedure was likewise done after the whole process of CDI experiments prior to the removal of electrodes from the plates. Likewise, the fresh and used electrodes were imaged in order to check for visible deterioration of the surface after CDI. The magnification for electrode defect imaging was 50× while 20× for contact angle measurement.

### 2.4. Chronoamperometry at Different Initial Influent Concentrations

Electrosorption at constant voltage of 1.2 V lasted for two hours. The desorption lasted for 30 min at 0 V. The influent initial concentrations used were 100 ppm, 2000 ppm, 15,000 ppm, and 30,000 ppm NaCl. Newly prepared electrodes were utilized for each run. The higher concentrations (2000 ppm, 15,000 ppm and 30,000 ppm) were selected to obtain an evident change in performance as the concentration approaches seawater concentration of at least 30,000 ppm from brackish concentration of at least 2000 ppm [9,26,27], while the lowest salt concentration (100 ppm) was used as the reference to compare the performance of CDI at higher concentrations tested. The electrode chronoamperometric performance was assessed using specific electrosorption capacity and charge efficiency. Specific electrosorption capacity (SEC) was calculated using Equation (1):

(1) $$SEC\;\left(mg\cdot g^{-1}\right)=\frac{M_{final}-M_{initial}}{M_{electrode}}$$

where *M_final_* and *M_initial_* are the final and initial mass of sodium chloride (mg) in the electrode respectively converted from the recorded charge on the electrode surface and *M_electrode_* is the mass of rGO/TiONTs in the electrode (g). Moreover, charge efficiency was calculated using Equation (2):

(2) $$charge\;efficiency\;\left(\%\right)=\frac{SEC}{\left(\int_{0}^{t}Idt\right)\left(\frac{M_{NaCl}}{F}\right)}$$

where *SEC* is the electrosorption capacity (mg·g^−1^), *I* is the specific current value (A·g^−1^) at time *t*, *M_NaCl_* is the molar mass of NaCl (58.44 g·mol^−1^), and *F* is Faraday’s constant (96,485 C·(mol e^−^)^−1^).

### 2.5. Chronocoulometry at Different Initial Influent Concentrations

Charge-discharge operation at constant current was set for CDI at different influent initial concentrations. This procedure immediately followed electrosorption-desorption at constant voltage for each level of concentration. Five different charging currents were used: 1, 0.5, 0.25, 0.125, and 0.1 A·g^−1^. The discharge current was maintained at −1 A·g^−1^, which aims to obtain an increasing charging-discharging time ratio. The performance was assessed based on the retention in charging time of the charging current for 5 cycles and the chronocoulometric or charge pattern on the electrode surface. Furthermore, to see the effect of repeated charge/discharge, a charging-discharging run was conducted with 100 ppm NaCl for 100 cycles with each cycle containing five different charging currents previously stated. 

The CDI module was connected to a potentiostat/galvanostat (BioLogic SP-150, Seyssinet-Pariset, France) that was used to control all the voltage and current. The maximum of the instrument in measuring all electrochemical parameters is only up to 0.1% of the full scale range. Flowrate was set at 25 mL·min^−1^ for all CDI operations. This instrument was connected to EC-Lab V10.40 software (Seyssinet-Pariset, France) that recorded all the data such as current, voltage, charge, and power consumption with respect to time.

## 3. Results and Discussion

### 3.1. Reduced Graphene Oxide/Titanium Dioxide Nanotubes Surface Properties

**Figure 1:**
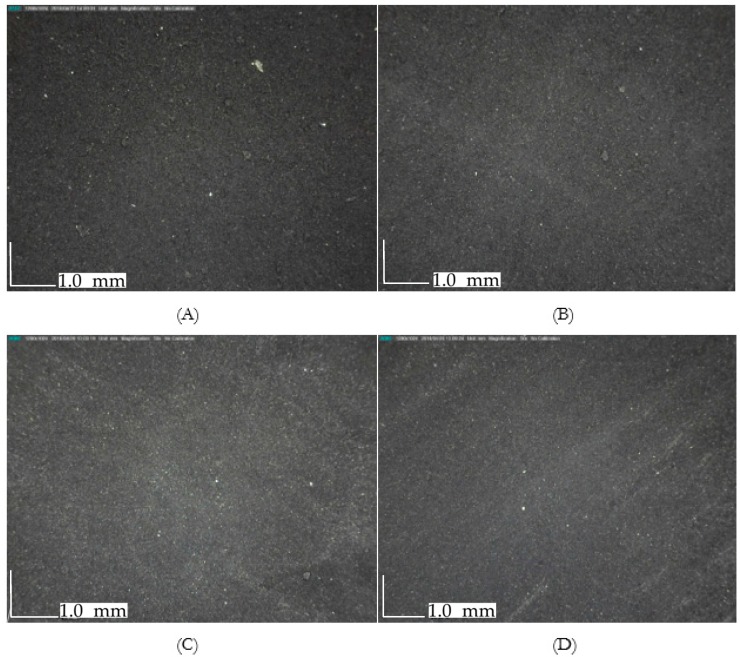
Magnified digital images of electrodes after operation at (**A**) 100 ppm, (**B**) 2000 ppm, (**C**) 15,000 ppm, (**D**) 30,000 ppm, showing an intact arrangement on the titanium current collector plate. No significant accumulation of sodium crystals on the surface were found. Magnification: 50×.

Figure 1 shows that the two-hour electrosorption, half-hour desorption and five-cycle charge-discharge did not alter the physical surface of the electrodes used at different concentrations. Aside from the apparently unaltered surface, its wettability did not change significantly. The mean contact angle measurement before CDI is 52.11°, which becomes 56.07° after CDI run. These two measurements were found under the region of good wettability as the contact angles for both tests were less than 90° [28].

The observed physical consistency in CDI material even after operation is a promising indication of its ability to withstand high influent concentration. However, its electrochemical response at varying concentrations would provide a deeper insight regarding this concern.

### 3.2. Chronoamperometry at Different Initial Influent Concentrations

**Figure 2:**
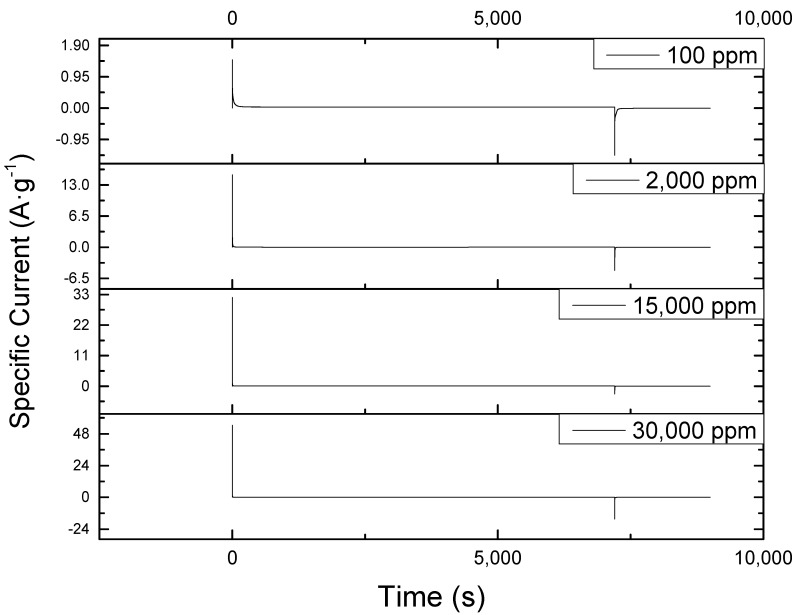
Chronoamperometric plot of CDI at different sodium chloride concentrations showing a consistent current behavior at all concentrations tested. The initial peak observed at the start of each of the operations is due to sudden introduction of 1.2 V to the system while the sudden drop towards the end of the reaction is fue to the removal of this voltage by the instrument, BioLogic SP-150.

The chronoamperometric plots at different concentrations of sodium chloride are shown in Figure 2. The smooth pattern indicated that the electrode coating defects and high concentrations did not hinder the process. Had the material been adversely affected by the high concentration, the current should keep fluctuating from a low to a high value and vice versa, but this kind of response was only observed at start and end of electrosorption process. Likewise, similar behavior was reported in the work of Tsai and Doong [29], Li et al. [6], and Gao et al. [12] involving low feed concentration.

Moreover, as seen from the plots, the curves after the peaks were only visible at 100 ppm. Thus, concentration is the main inducer of electrosorption to the surface of the material. The greater the concentration of NaCl, the greater the number of ions will be available for electrosorption.

The positive effects of an increased concentration on the operation was further illustrated by examining its effect to the initial charging and discharging current at constant voltage of 1.2 V. As reported in Table 1, it was found that the higher the concentration, the higher the magnitude of the initial charge and discharge current. The inconsistency found at an initial discharge current of 15,000 ppm run was due to the instrument’s failure to record the current at exactly 7200 s (two hours). Thus, it was not able to record the peak discharge current. Moreover, the absolute ratio of initial charge to discharge current tends to increase with increasing NaCl concentration. Thus, the number of ions attracted by the charged electrode during electrosorption is more than the number of ions returned to the solution during desorption at highly concentrated NaCl solutions. 

**Figure 3:**
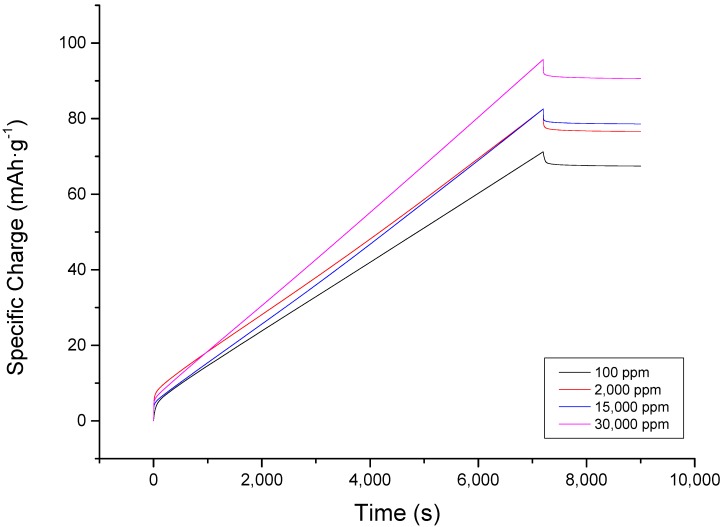
Charge accumulation plot at different concentrations of sodium chloride showing a consistent trend throughout the electrosorption and desorption stage. Rate of specific charge accumulation increases with concentration as shown by the slope. The initial specific charge accumulated, however, does not correlate to the initial concentration but due to uncontrollable factors caused by the instrument and set-up.

The aforementioned behavior may be beneficial in terms of electrosorption. However, this may not be helpful when the material will be used for a prolonged operation as it will require recycling. To illustrate the trade-off between the charging capability and discharging deficiency observed with the chronoamperometric plot, the charge accumulated had to be examined as well. Figure 3 shows the charge accumulation with respect to time for different NaCl solutions influent concentrations, and it shows that even at two hours, the electrode has not been saturated. It is evident from the plot that there was an initial immediate spike of charge accumulated on the plates. Since the instantaneous charge accumulated at the start of operation cannot be completely controlled due to automated current regulation by the instrument and minute differences in electrode thickness during coating that may have affected initial reaction of the ions to incoming electric field, Figure 3 must be analyzed in terms of its rate of charge accumulation upon reaching a stable operation, rather than the absolute charge accumulated at any given time. Sodium chloride (expressed in units of charge) accumulated at different rates, but for all concentrations, the accumulation was linear. The steepest slope was observed at 30,000 ppm. The slope becomes less steep when the concentration is lowered. This meant that at the end of two hours, those of lower slopes reached a lower ion accumulation. Therefore, even though the behaviors of the desalination of 2000 and 15,000 ppm NaCl are similar in terms of specific charge accumulation, the difference in slopes affirms that desalination by CDI is improved when influent concentration is increased. On the other hand, during discharge, after a sudden dip in charge, the plot almost reached an equilibrium value. In 30 min, the line is already almost asymptotic.

While the charging capacity of the material improved at higher concentration, discharge phase behaved differently. The percent discharge, which was found to be independent of concentration, ranged from 4.9–7.27% as reported in Table 2. This means that the problem of ion discharge is not to be associated with the inability of the material to desorb ions due to the concentration level, but with the inability of the zero voltage discharge operation to reverse the electrosorption as compared to an operation with reversed polarity or current [30].

Although it has already been established that electrosorption in 3:1 rGO/TiONTs is induced by concentration, the value of specific electrosorption capacity of the composite has not been reported up to this point. Table 3 reports increasing electrosorption capacity by the material starting at 100 ppm. However, its charge efficiency slightly decreases with an increase in concentration. Furthermore, the specific energy consumption is directly proportional to its associated electrosorption capacity. The same behavior was reported in the work of Y. Zhao et al. [31] and Dementzis & Wessner [32]. The increasing energy requirement was expected since more and more ions are pushed to the inner layer near the material when concentration is increased. However, the ratio of the energy consumption and electrosorption capacity remains constant at 1.11 kWh·kg^−1^. With the same amount of energy consumed per mass of salt removed, a concentration of 30,000 ppm is advantageous as the electrode was able to capture more salts.

Aside from the fact that electrode performance is enhanced at higher concentrations, rGO/TiONTs outperforms the materials synthesized in recent times. Table 4 shows that the sodium chloride removal of reduced graphene oxide/titanium dioxide nanotubes composite performance compared to other materials synthesized as reported in the literature. The electrosorption capacity of the composite used in this study is evidently better than those reported in the literature even with its lowest electrosorption in this study obtained at 100 ppm NaCl. This excellent performance is attributed to the highly capacitive and polar surface of the composite caused by the mesoporous arrangement attained by rGO and TiONTs at 3:1 mass proportion together with the functional groups present in each component [7,8].

### 3.3. Chronocoulometry at Different Initial Influent Concentrations

Aside from the constant voltage CDI that highlights the material’s superior electrosorptive performance, constant current CDI provides additional insights into the ability of the material to be used sustainably. 

**Figure 4:**
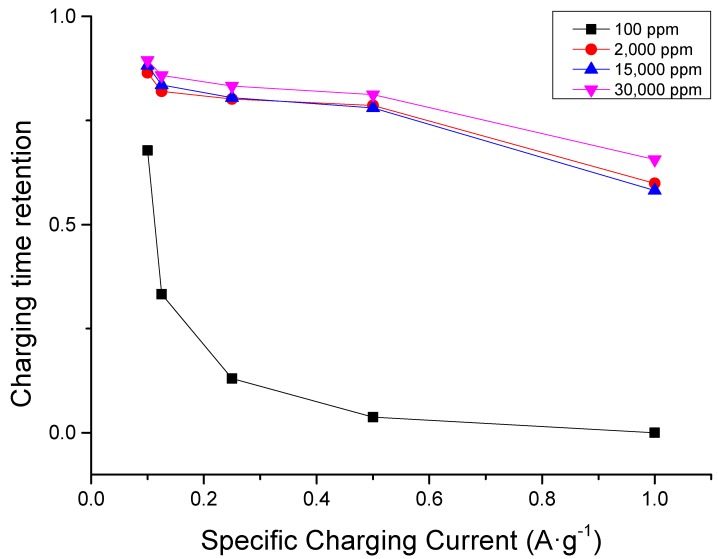
Charging time (capacity) conservation plot at different sodium chloride concentrations showing that retention increases with increasing concentration. Lower specific charging current leads to a higher retention due to more rigorous and thorough ion removal during discharge.

As illustrated in the chronocoulometric plot of operation at different concentrations found in Figure 4, charging time retention is decreased for all concentrations when specific charging current is increased. Moreover, the decrease in retention is less pronounced when the concentration is higher. This implies that a higher concentration either helps preserve electrode quality or gives a higher ion availability of for electrosorption. The latter seems more logical because the order of cycle begins with the fastest charging current (1 A·g^−1^) and ends with the slowest charging current (0.1 A·g^−1^). 

**Figure 5:**
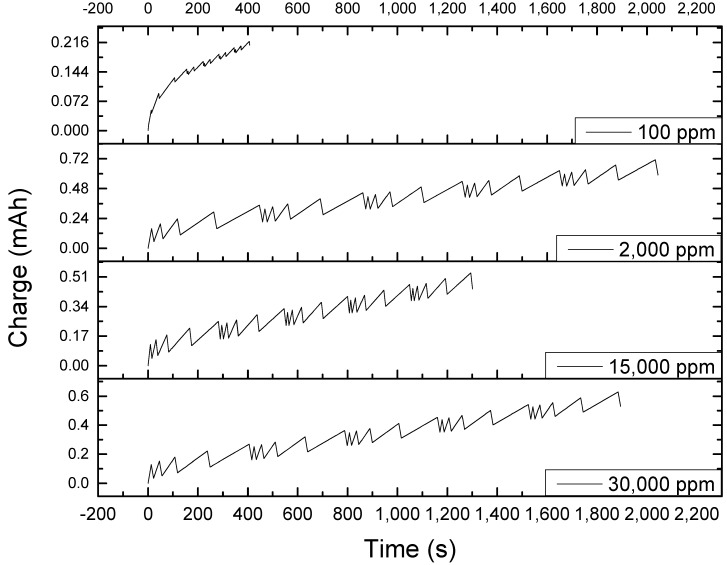
Chronocoulometric plot at different sodium chloride concentrations set at five different charging currents repeated five times per set, showing that the electrodes were not completely discharged every end of the cycle, leading to a net accumulation of charge as the cycle is repeated each set.

If the electrode were already spent by the middle of the cycle, then lower charging currents should have an even faster charging time, but the operation always had an increasing time consumed with the lowering of the specific charging current as shown in Figure 5.

A deeper understanding of the behavior recently discussed could be provided by the operation’s chronocoulometric or charge plot. The chronocoulometric plot in Figure 5 shows the recurrence of a pattern every five peaks, with each peak having a wider base as the cycle progresses. Theoretically, it is expected that each charge-discharge start and end at complete discharge. However, it was discovered from the chronocoulometric plot that discharge was not complete. This caused the lowering of charging time as the cycle progressed but, compared to the discharge inefficiency observed with zero-voltage desorption, the performance of this operation was much better. Moreover, this plot shows that the charging time was significantly shorter for low sodium chloride concentration. These observations illustrate that the low retention obtained at repeated charge-discharge does not necessarily mean that the electrodes degenerate. Rather, the apparent decrease in its usability is due to the ions that fail to be discharged completely. This can be solved by changing the operational conditions of the system. Rather than controlling the voltage, the charge should be controlled instead. However, this may lead to a voltage greater than 1.2 V at discharge, something that should be avoided because beyond this voltage, electrolysis of water and oxidation of titanium plates can happen. Thus, a more viable solution would be to use a slower charging current when performing repeated cycles.

**Figure 6:**
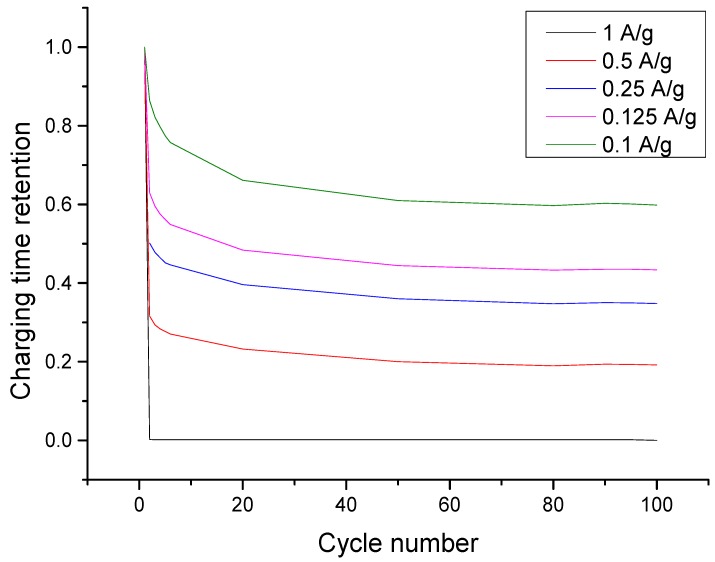
Charge time (capacity) conservation plot at different charging currents showing that retention increases with specific current. All plots approach a lower limit of charging time retention with increasing number of cycles, implying that the electrode can be recycled for at least 100 cycles.

To attain a deeper insight into how charge accumulation lowers the retention of the charging time (capacity) of the electrodes, the lowest concentration was further tested for a prolonged constant current charge-discharge capacitive deionization. As shown in Figure 6, the retention plot of each charging current, it was evident that retention was lowest at 1 A·g^−1^ and highest at 0.1 A·g^−1^. This means that a slower charging rate can maximize the remaining capacity of the electrode compared to a faster charging rate. This can likewise explain the observation that the charging time retention is lowest when the current applied is 1 A·g^−1^. Since slow charging at 0.1 A·g^−1^ accumulated more charge than the rest, when it was discharged at −1 A·g^−1^, only few ions were desorbed compared to what has been accumulated. This further suggests that a slower discharge time might facilitate a better consistency in terms of restoring back the total capacity of the electrode. Furthermore, as the charging time retention plot is asymptotic as it approaches 100 cycles, the 3:1 rGO/TiONTs composite helps in making desalination by CDI sustainable as it would not require the frequent replacement of spent electrodes.

## 4. Conclusions

The synthesized composite of reduced graphene oxide/titanium dioxide nanotube at 3:1 parts by mass was found to have great potential as an electrode for capacitive deionization in order to perform desalination up to concentrations as high as 30,000 ppm of NaCl. The performance of the composite was assessed in terms of its chronoamperometric, chronopotentiometric, and chronocoulometric response. Capacitive deionization of NaCl at different concentrations using constant voltage (chronoamperometric) and constant current (chronopotentiometric) gave desirable results. Data gathered showed that 3:1 rGO/TiONTs is capable of recovering salts from water, showing stability even at higher concentrations. It was able to attain a high electrosorption at low energy consumption. Moreover, it was verified that decrease in retention was not a material failure, but an operational failure that led to incomplete desorption of sodium chloride ions. Hence, the material is robust, making it a good material for sustainable use as there is no need to dispose of it frequently. Furthermore, the obtained result is a good indicator that large-scale desalination with 3:1 rGO/TiONTs used in this study will require less electrode compared to other available materials for desalination by CDI. This would translate to lowering of fixed cost brought about by the equipment size and to lowering of variable cost due to lower amount of energy and less frequent electrode replenishment needed to attain the desired water quality. Moreover, due to the simplicity of the microwave reaction method used to combine rGO and TiONTs, the composite can be mass-produced not only for desalination or other water treatment/purification requirements, but also for other purposes requiring highly capacitive materials such as energy storage in batteries, capacitors and supercapacitors.

One drawback experienced in this study was the inability of the material to discharge completely at zero voltage. While it may not be possible to improve desorption of salts using 3:1 rGO/TiONTs composite, its performance as an electrode for sustainable desalination may still be enhanced by using a low discharge current to maximize the removal of ions from the electrode. This would allow greater retention of electrodes when used repeatedly. Moreover, in future experiments, discharge should not be at zero voltage because such setting would not reach 8% removal of adsorbed ions. A slow charging current is likewise a good consideration if the goal is to increase the total electrosorption per cycle. Prolonging the electrosorption phase is further encouraged in order to reach saturation. This will maximize the capacity of the material and provide deeper insights into the kinetics of the operation.

## Figures and Tables

**Table 1:** The effect of initial influent concentration to the initial charge-discharge current ratio.

Initial Concentration (ppm)	Initial Charge Current (A·g^−1^)	Initial Discharge Current (A·g^−1^)	Absolute Ratio
100	1.47	−1.42	1.04
2000	15.15	−4.82	3.14
15,000	31.97	−2.85	11.22
30,000	54.44	−16.40	3.32

**Table 2:** Percent discharge at different initial concentrations of influent sodium chloride solutions.

Initial Concentration (ppm)	Charge after Electrosorption (mAh·g^−1^)	Charge after Desorption (mAh·g^−1^)	Percent Discharged (%)
100	71.11	67.5	5.08
2000	82.50	76.5	7.27
15,000	82.55	78.51	4.90
30,000	95.56	90.56	5.23

**Table 3:** Specific electrosorption capacity, charge efficiency and energy consumption of 3:1 rGO/TiONTs at different initial concentrations of influent sodium chloride solutions.

Initial Concentration (ppm)	rGO/TiONTs Mass (mg)	Specific Electrosorption Capacity (mg Sodium Chloride Per g Electrode)	Charge Efficiency (%)	Specific Energy Consumption (×10^6^, kWh·g^−1^)	Energy Consumption Per Kilogram of Sodium Chloride Removed (kWh·kg^−1^)
100	36	77.66	99.96	86.11	1.11
2000	40	89.98	99.53	99.54	1.11
15,000	47	90.00	99.26	99.88	1.11
30,000	36	104.29	98.46	116.61	1.12

**Table 4:** Sodium chloride electrosorption capacity of different materials available in the literature in comparison to 3:1 reduced graphene oxide/titanium dioxide nanotubes composite studied.

Electrode	Initial Concentration (ppm or mg·L^−1^)	Flow Rate (mL·min^−1^)	Time (min)	Electrosorption Capacity (mg·g^−1^)	Reference
TiO_2_ nanorod-rGO	280	20	5	9.1	[11]
Mesoporous carbon	500	34	180	4.8	[12]
Carbon black loaded N-doped carbon aerogel	1461 (25 mM)	Not reported	120	7.3	[13]
Fe_3_O_4_ etched mesoporous graphene	150 (300 µS·cm^−1^)	10	120	10.3	[14]
Highly porous activated carbon	584.4 (10 mM)	5	60	22.5	[15]
Graphene oxide/auricularia	55.72	Not reported	56	7.74	[16]
Granular activated carbon	1649 (1000 ppm Cl^−^)	168	300	9.6 (5.8 mg Cl^−^·g^−1^)	[17]
Activated carbon	1168.8 (20 mM)	12	Not reported	12.1	[18]
Hollow zeolitic imidazolate frameworks derived nanoporous carbon	500	25	40	15.31	[19]
Polyaniline activated carbon	600	26	56	15.3	[20]
Graphene hydrogel	500	10	300	49.34	[21]
P-doped carbon nanofiber aerogels	1649 (1000 ppm Cl^−^)	50	60	16.20	[22]
3:1 Reduced graphene oxide/titanium dioxide nanotubes	100	25	120	77.66	This work
